# Measles and Pregnancy: Immunity and Immunization—What Can Be Learned from Observing Complications during an Epidemic Year

**DOI:** 10.1155/2020/6532868

**Published:** 2020-08-01

**Authors:** Rosalia Ragusa, Armando Platania, Mario Cuccia, Gaetano Zappalà, Gabriele Giorgianni, Placido D'Agati, Maria Alessandra Bellia, Marina Marranzano

**Affiliations:** ^1^Health Technology Assessment Committee-University Hospital “G. Rodolico”, Via Santa Sofia, 78, 95123 Catania, Italy; ^2^Department of Medical and Surgical Sciences and Advanced Technologies “G.F. Ingrassia”, University of Catania, Via Santa Sofia 87, 95123 Catania, Italy; ^3^Epidemiology and Prevention Service, ASP 3, Provincial Agency of Health of Catania, Via Tevere 32, San Gregorio di Catania, Italy; ^4^School of Medicine, University of Catania, Via Santa Sofia, 78 95123 Catania, Italy

## Abstract

Measles is a highly contagious airborne disease. Unvaccinated pregnant women are not only at risk of infection but also at risk of severe pregnancy complications. As measles causes a dysregulation of the entire immune system, we describe immunological variations and how immune response mechanisms can lead to adverse pregnancy outcomes. We evaluated data during the measles outbreak reported in the province of Catania, Italy, from May 2017 to June 2018. We controlled hospital discharge records for patients admitted to hospital obstetric wards searching the measles diagnostic code. We have indicated the case as “confirmed” when the IgM was found to be positive with the ELISA method. We registered 843 cases of measles and 51% were females (430 cases). 24 patients between the ages of 17 and 40 had measles while they were pregnant. Adverse pregnancy outcomes included 2 spontaneous abortions, 1 therapeutic abortion, 1 foetal death, and 6 preterm deliveries. Respiratory complications were more prevalent in pregnant women (21%) than in nonpregnant women with measles (9%). 14 health care workers (1.7%) were infected with measles, and none of these had been previously vaccinated. Immune response mechanisms were associated with adverse pregnancy outcomes in women with measles. To reduce the rate of measles complications, gynaecologists should investigate vaccination history and antibody test results in all women of childbearing age. During a measles outbreak, gynaecologists and midwives should be active proponents of vaccination administration and counteract any vaccine hesitancy not only in patients but also among health care workers.

## 1. Introduction

Measles is a highly contagious airborne disease though the virus has brief persistence in the environment. According to a recent report of the World Health Organization (WHO), approximately 110,000 people died from measles in 2017, mostly children under the age of 5 years, despite the availability of a safe and effective vaccine [1]. Unvaccinated pregnant women are not only at risk of infection but also at risk of complications in pregnancy. Immune response mechanisms may interfere with the normal course of pregnancy. A key public health strategy, to limit the global burden of measles and measles-related deaths, should include routine measles vaccination for children, combined with mass immunization campaigns in countries with high incidence and death rates associated with measles. Prevention through immunization is highly protective; it is 93% effective at preventing measles after the administration of the first dose and 97% effective after the second dose. The duration of protection is estimated to be lifelong. [2]

In the United States, measles was declared eliminated in 2000. Since 2016, the annual number of imported cases (28) has increased; overall, 1077 cases have been reported in the first six months of 2019 which is the highest number of reported cases since 2000 [3]. In August, the numbers continue to increase, and CDC reported the highest number of cases since 1992 [4].

Although the vast majority of cases worldwide occur in countries with weak health systems, large outbreaks of measles also have been reported in developed countries indicating a return of the dangerousness of the disease; this is occurring in areas where effective vaccination programs were present for decades. These comebacks can be explained by immunity gaps in the population [5, 6] and vaccine refusal. This is emerging as a risk factor for measles outbreaks, and the WHO has identified vaccine hesitancy as one of the top 10 global health threats in 2019 [7].

In Italy, measles vaccination has been recommended since 1976, and the current goal of the Ministry of Health is to vaccinate at least 95% of children within two years of age. However, up until the mid-1990s, the vaccination coverage did not exceed 60%, and in 2000, it was 70%. In 2017, a large outbreak occurred in Italy, especially in Sicily, a large region in the Southern part of Italy. Measles usually has the highest incidence among 5–9-year-olds, who generally accounted for more than 50% of the reported cases [8]. The peculiar characteristic of this outbreak was a shift in the median age to 22 years old, with a particular involvement of young adults, including pregnant women.

The measles virus is not teratogenic; however, it alters the physiological mechanisms of immunotolerance, present during pregnancy, through changes that mainly involve cell-mediated immunity. This can lead to a reaction similar to rejection manifested by spontaneous abortion or premature expulsion of the foetus.

The aim of this study was to present epidemiological data and describe the immunological variations and complications during an outbreak of measles. Secondary aim was to investigate potential interventions that could be used to reduce the rate of measles complications, taking into account that vaccination cannot be performed during pregnancy.

## 2. Methods

### 2.1. Data Collection

We collected information on measles cases reported to the Provincial Agency for Health of Catania, Sicily, Italy, during the measles epidemic period between 2017 and 2018. Catania is the second largest province of Sicily with approximately 1100,000 inhabitants. It is a part of the southern Italy region where the highest number of measles cases was recorded in 2018. The data in the province of Catania have been compared with the data from the Sicilian region (5 million inhabitants) and data from all Italy (60 million inhabitants). These comparative data were provided by the National Institute of Statistics (ISTAT) and analysed to better understand the progress of the epidemic.

We extrapolated data on women of childbearing age (17-40) to verify the incidence and the recorded complications of measles among these individuals. We analysed the data to determine measles incidence during pregnancy and associated outcomes including complications.

Three data sources were used:

1. Cases reported to the Complex Operating Unit “Epidemiology and Prevention” of the Provincial Agency for Health of Catania (ASP 3) from May 1, 2017, to June 30, 2018
2. Hospital discharge records of patients admitted to hospital obstetric wards in the province of Catania during the period considered. We used the International Classification of Diseases, 9th Revision, Clinical Modification (ICD-9-CM) to search for the following diagnostic codes:

*(i) Code 055.0-55.9*. Measles and subcategories with or without mention of complications

*(ii) Code 647.63*. Other viral diseases in the mother, antepartum condition, or complications

*(iii) Code 647.61*. Other viral diseases in the mother, who delivered, with or without mention of antepartum condition

Patient medical records were further collected to retrieve medical history and pregnancy outcomes and complications. Data were then collected and coded in a single database.

(3) Data from the ISTAT were consulted for the verification of regional and national data

To confirm the diagnosis, a blood sample is taken. The serum is measured for measles-specific immunoglobulin M (IgM). Serologic testing is done by enzyme-linked immunoassay (ELISA or EIA) for IgM antibodies.

### 2.2. Statistical Analysis

We did a descriptive analysis due to the small sample size. We compared the rates of each complication between pregnant and nonpregnant women. The threshold for significance was a *p* value less than 0.05. Statistical analyses were performed using the Statistical Package for Social Sciences (SPSS) version 20.0 (SPSS Inc., Chicago, IL, USA).

## 3. Results

In 2017, a large measles outbreak occurred in Italy from January 1 to December 31, 2017; during this time, 5393 measles cases were reported. The incidence of measles increased from 1.4 cases/100,000 inhabitants in 2016 to 8.2/100,000 in 2017 indicating a serious outbreak. In 2018, with 2526 measles cases reported in Italy, the incidence fell again to 4.2/100,000.

In Sicily, the outbreak spread later than in Italy, and the peak was recorded in 2018 and consisted of approximately half of all reported national cases. We have gone from a measles incidence rate of 1.3/100,000 in 2016 to 8.4 cases per 100,000 in 2017 reaching 22.2/100,000 during 2018.

We registered 425 cases in 2017 (7.8% of the national total) including 4 deaths. Overall, 81% of the cases in 2017 were confirmed by laboratory tests with specific IgM positivity results. Those most affected included individuals over 15 years of age, and 51% were females. Deaths associated with measles occurred due to complications, especially involving the respiratory system.

In 2018, 1117 cases (46% of the national total) were reported in Sicily, and 74% of cases were confirmed by laboratory tests in 2018. The median age of those infected was 25 years, and 47% of the cases reported at least one complication. So far, 8 deaths have been reported, and 115 cases were reported among health care professionals.

Similar to other Sicilian provinces, the outbreak in Catania occurred mainly between May 2017 and June 2018, and during this period, 843 cases were reported. All cases were confirmed by anti-measles IgM positivity. The median age of those affected was 22 years, and 51% were females (430 cases).

Among the 843 cases reported, overall, 14 health care workers (1.7%) were infected with measles, and none of these had been previously vaccinated.

A total of 24 cases of measles during pregnancy were registered. The clinical features of the individual cases are reported on Table 1.

The women were 17 to 40 years of age (median 27). All pregnant women affected by measles presented fever and rash at a distance of between one and six days from fever. Among all cases, there were 2 spontaneous abortions (5 and 15 weeks of gestation), and one therapeutic abortion was reported at the eleventh week. An intrauterine foetal death occurred at the thirty-third week of gestation. After spontaneous delivery, caesarean section was performed. The foetus presented the umbilical cord looped around the neck. The mother presented stomatitis, keratoconjunctivitis, laryngitis, tracheitis, bronchitis, pneumonia, and respiratory failure. Six preterm deliveries (4 premature deliveries, 28-32 weeks of gestation, 2 preterm deliveries, 32-37 weeks of gestation) and 13 term deliveries occurred.

Twenty patients were hospitalized due to measles or relative complications. GD was hospitalized at the twenty-fifth and thirty-fourth weeks of gestation and gave birth at the fortieth week reporting the complications described in the table.

The frequency of measles complications was calculated for women with measles during pregnancy (*n* = 24) and for nonpregnant women of the same age group with measles (*n* = 227) (17-40 years) of the 843 total cases of measles. During the follow-up, after diagnosis of measles, we lost only a pregnant woman. She was excluded from the overall evaluation. However, although the sample size is small, the frequency of respiratory complications between pregnant and nonpregnant women was statistically significant (Table 2).

In both groups, none of the women who developed pneumonia or respiratory failure had symptoms of underlying diseases as asthma or bronchitis.

## 4. Discussion

All six WHO regions have the goal of measles elimination by or before 2020 [9], and regional elimination is a step on the path toward global eradication. While progress has been made in these fields, the regional elimination goal has not been achieved [10].

The large outbreak of measles with the characteristic age shift that occurred in Italy in 2017 has gained more attention from epidemiologists and other clinicians rather than only paediatric specialists. The disease notification data show that a predominance of forms (79.5%) was filled out in hospitals or emergency rooms while the minority (20.5%) were filled out by paediatricians and general practitioners in private clinics. It must be stressed that the actual number of cases is more likely to be at least 2-3 times higher due to the reduced notifications of cases managed at home.

This study focused on pregnant women with measles to give an overview of the complications and outcomes associated with measles infections in this special group of patients. Our results demonstrate that the spontaneous abortion rate (8%) aligns with the frequencies observed in the general population [11, 12]. Nevertheless, if we consider all adverse pregnancy outcomes, including 1 foetal death and 1 therapeutic abortion (due to the apprehension of the woman regarding possible newborn's health problems associated with measles), the impact of the disease on pregnancy outcomes is significant (16% of pregnant women had adverse outcomes).

The evidence regarding the association of measles in pregnancy and spontaneous abortion is limited; however, a recent review found higher rates of pregnancy loss in pregnant women with measles, especially in developing countries [13, 14]. The preterm delivery rate in our study was higher (25%) than that of the general population (which as varied from 5% to 15%) [15]. In addition, 4 of the 6 preterm births took place before the 32 weeks of gestation, which can have detrimental consequences for the newborn including lengthy stays in the intensive care unit. Previous studies have also suggested that measles in pregnancy can increase the frequency of preterm deliveries as well as low birth weight [16, 17].

Measles in pregnancy can also lead to perinatal infections in the newborn, which can be associated with a high mortality and neurological complications such as subacute sclerosing panencephalitis [18]. Thus, it is recommended that susceptible pregnant women exposed to measles receive 1 g of human normal immunoglobulin (Ig) within 72 h and not more than 6 days of exposure to prevent or modify the course of the disease.

Another aspect of measles in pregnancy concerns its direct effects on pregnant women. Mortality and morbidity risks associated with measles in pregnant women are higher than in nonpregnant women. In particular, pregnant women with measles are more likely to be hospitalized, to develop pneumonia, and to die when compared to nonpregnant women with measles [19].

In our study, a high rate of pregnant women developed respiratory complications, including pneumonia, while nonpregnant women affected by the same measles outbreak had a significantly lower rate of respiratory complications. Uninfected pregnant women are more likely to develop minor complications that often do not require hospitalization such as stomatitis and conjunctivitis.

Immune response mechanisms could be responsible for the adverse pregnancy outcomes in women with measles. Those infected with measles can produce an efficient and effective immune response directed by T lymphocytes that aim to eliminate the virus from the body. Overall, CD8 levels increase, and a maculopapular rash develop, as a consequence of the interaction between T lymphocytes and infected cells [20].

Therefore, the integrity of cell-mediated immunity is essential to cope with measles infections. A strong immune response is set up by the organism against the virus and, after the onset of the exanthema, a nonspecific and temporary immunosuppression is developed. This immunosuppression is in part responsible for the susceptibility to other infections. In addition to immuno-depression, measles infections lead to a dysregulation of the entire immune system, indicated by a characteristic increase in IgE.

In children with impaired cellular immunity, the infection has an unfavourable course due to complications such as giant cell pneumonia or encephalitis. However, in the agammaglobulinemic patients, the infection follows its usual course with the development of a secondary immunity without the production of Ig antibodies.

The association between the immune response and the possible mechanisms underlying the failure of immune-mediated responses during pregnancy has been described [21]. In women, the endometrium can be considered a tertiary lymphoid organ, whose cellular composition changes in the various phases of the menstrual cycle and during pregnancy [22]. At the placental level, important modifications of the immune response occur, conditioned by the different modalities of contact between the cellular constituents of the mother and the foetus [23].

During the first trimester of pregnancy, the subpopulation of natural killer uterine cells (uNK), a specialised and specific cellular subset of the uterus, increases to comprise approximately 70% of the leukocyte population in the endometrium. The uNK cells intervene in the complex regulatory mechanisms that govern the early implant phases and the placentation phenomena through the secretion of various cytokines. The panel of cytokines produced by uNK cells carries out different “sign” actions towards the product of conception, and the final outcome of pregnancy depends ultimately on a fine regulation of the “cocktail” of cytokines produced [24, 25, 26, 27]. Therefore, pregnant women have additional risks, due to the particular immune response.

The only effective measure to avoid measles in pregnancy is to improve the adherence of the general population to vaccination programs [ 28]. In Sicily, on July 31, 2017, mandatory vaccination against measles, mumps, and rubella was introduced for children under the age of 16 in order to increase vaccination coverage currently below the minimum threshold of 95% and to obtain the protection of individuals and the “immunity of the population” within a population.

Measles have evolved from a disease contracted in paediatric age groups to the creation of pockets of unvaccinated populations which has manifested a disease that affects a broad range of young adults. To protect young women who plan to get pregnant, the guidelines established by the WHO must be implemented because MMR vaccines are contraindicated during pregnancy. For this reason, within a measles outbreak, it is advisable to verify a woman's susceptibility to measles, especially among those who wish to get pregnant, in order to promptly administer the vaccine.

Vaccine hesitancy is spreading, not only among citizens but also among healthcare workers (HCWs) with a consequent steady reduction in vaccine coverage [29]. It should be noted that among the 24 women of the study, one was a health care worker who had therefore not followed the recommendation to be vaccinated and chose therapeutic abortion for fear of possible damage for the unborn child. This overall general hesitancy among citizens and health care workers can affect fragile populations including newborns and pregnant women. We identified 14 health care workers with measles who were not vaccinated despite the 2017-2019 Italian National Immunization Prevention Plan that strongly recommended health care workers to be vaccinated [30]. HCWs are exposed to biological hazards with daily work. The implementation of strict prevention measures on HCWs can reduce the possibility of contracting the virus in the workplace and passing the infection on to other patients and to healthcare staff, as indicated in other biohazard situations for them [31, 32]. Among the suggested measures and the ones considered effective, we reminded the possibility of requesting the mandatory vaccination in operators working in delicate departments such as gynaecology, neonatology, paediatrics, or neonatal intensive therapy. The synergistic action of influential medical professionals and communication training on counselling techniques that can be applied to all health workers is mandatory.

As it is already acknowledged among health professionals the validity of exclusive breastfeeding in the first months of life, it must also be supported by health worker's vaccination against measles [33].

Gynaecologists and midwives have an important role in reducing complications: the introduction of routine testing to assess measles susceptibility may allow to make decisions about the possibility of reducing the complications of the disease in the newborn.

In case of prepregnant woman, they may propose vaccination options.

Dosing antibodies during pregnancy and thus, know the immunological situation of the mother, may, in the case of a measles epidemic, suggest that the first dose of vaccine be given in advance to protect the infant [34].

The main limit of the study is the following: it is a single-centre study, and therefore, a much larger study would be required to draw stronger conclusions. However, it is important to highlight that it is not possible to accept serious complications, such as those described, for a disease that can be eradicated. Even if there could be only a small number of complications, it is necessary to do whatever it takes to avoid the spread of other epidemics and the possible consequences.

## 5. Conclusions

This study presented the immunological variations that explain the severity of measles infection, whether contracted by pregnant women or in newborns.

Measles in pregnancy should not be underestimated, especially in an outbreak situation, as it can increase the risk of both maternal and newborn morbidity and mortality.

The measles virus is not teratogenic; however, it alters the physiological mechanisms of immunotolerance during pregnancy through changes that mainly involve cell-mediated immunity. This can lead to a rejection-similar reaction seen with spontaneous abortion or premature expulsion of the foetus. The possible increased rates of adverse pregnancy outcomes and maternal complications must be considered. It is hoped that early detection of the disease and an appropriate patient management can reduce complications.

Since measles vaccines are contraindicated in pregnancy, targeted vaccination programs for susceptible people are mandatory: adolescents, young women who plan to get pregnant, and health care workers.

Gynaecologists and midwives have an important role in reducing complications: the introduction of routine testing to assess measles susceptibility may allow to make decisions about the possibility of reducing the complications of the disease on the newborn.

## Figures and Tables

**Table 1:** Clinical features and outcome of 24 cases of women with measles in pregnancy (V: vaginal delivery; C: caesarean section; A: abortion/miscarriage).

Case	Symptoms of measles																
	Case	Age of the mother (years)	Pregnancy number	Fever	Rash	Rhinitis	Conjunctivitis	Cough	Adenopathy	Arthralgia	Arthritis	Gestational age at the time of admission for measles (week)	Gestational age at the time of delivery (week)	Type of birth	Outcome	Complications of measles	Notes and discharge diagnosis
MA	1	26	2	x	x			x				32	38	V	Live birth	No	
MS	2	19	1	x	x		x	x				32	33	C	Stillborn	Pneumonia, respiratory failure, stomatitis, intrauterine death	Umbilical cord wrapped around the neck, premature childbirth
LF	3	38	1	x	x		x	x	x			39	39	V	Live birth	Keratoconjunctivitis, pneumonia, stomatitis	
LA	4	37	2	x	x			x	x			15	15	A	Miscarriage	Pneumonia, diarrhea	Fibrosing alveolitis, dyspnea
FN	5	22	2	x	x			x	x			28	29	C	Live birth	Pneumonia, diarrhea, stomatitis, keratoconjunctivitis	Threat of premature labor
NF	6	24	1	x	x		x	x	x			11	11	A	Abortion	No	Blood loss, voluntary abortion
TA	7	18	2	x	x		x		x			26	28	C	Live birth	No	Premature childbirth
LM	8	23	2	x	x		x	x				16	38	V	Live birth	No	
FG	9	29	1	x	x			x	x			19	39	C	Live birth	Liver dysfunction	Oligoidramnios, foetal distress
PC	10	17	1	x	x								39	V	Live birth	No	
TS	11	35	3	x	x								39	C	Live birth	No	Threat of premature labor
PC	12	28	2	x	x								41	V	Live birth	No	
UF	13	30	1	x	x								38	C	Live birth	No	Abnormalities of heart rate or of fetal heart rate
GA	14	30	1	x	x			x		x		6	32	V	Live birth	No	Chronic hepatitis
PA	15	34	1	x	x	x		x	x			28	32	C	Live birth	No	Premature rupture of membranes, diabetes mellitus, insufficient foetal development
RA	16	25	1	x	x			x				36	36	C	Live birth	Pneumonia	Hypertension, fever
DG	17	28	1	x	x							31	38	V	Live birth	No	
SM	18	26	1	x	x							24	38	V	Live birth	No	
MC	19	39	2	x	x	x	x		x			19	Lost to follow-up	/	Lost to follow-up	Thrombocytopenia	
LL	20	40	2	x	x		x	x	x			4	Miscarriage	A	Miscarriage	No	Voluntary abortion
VL	21	24	1	x	x	x	x		x			38	38	C	Live birth	Pneumonia	
FD	22	23	1	x	x			x	x			26	38	C	Live birth	No	
MV	23	29	2	x	x			x	x			33	33	C	Live birth	No	Premature release of placenta, blood loss antepartum
GD	24	22	1	x	x	x	x	x				25 (1st)	40	V	Live birth	Threat of premature labor	
												34 (2nd)				Hypertension, preinduction with prostaglandin, short umbilical cord, umbilical cord around the neck, mild preeclampsia proteinuria, increase of uric acid, hypoalbuminemia. Therapy: albumin, antibiotics, antithrombin III	Right renal pelvic dilatation at 6th month, hypertension, hypoalbuminemia, haemoglobinuris and proteinuria, bilateral edema, no gasps

**Table 2:** Frequencies of measles complications in pregnant women with measles versus nonpregnant women with measles.

Women with measles—age 17-40 (*n* = 251)			
	Pregnant (*n* = 24) 9.6%	Nonpregnant (*n* = 227) 90.4%	*p* value
Complications	*n* (%)	*n* (%)	
Pneumonia	6 (25)	20 (8.8)	0.013
Otitis	0 (0)	4 (20.4)	0.51
Keratoconjunctivitis	2 (8.3)	66 (29.1)	0.02
Croup	1 (4.1)	12 (5.3)	0.81
Diarrhoea	2 (8.3)	35 (15.4)	0.35
Stomatitis	3 (12.5)	44 (19.4)	0.41
Convulsions	0 (0)	2 (0.9)	0.64
Hepatitis	1 (0)	43 (18.9)	0.06
Thrombocytopenia	1 (4.1)	13 (5.7)	0.75
Encephalitis	0 (0)	0 (0)	/

## Data Availability

All data are available if requested.
